# Detection of lipid-induced structural changes of the Marburg virus matrix protein VP40 using hydrogen/deuterium exchange-mass spectrometry

**DOI:** 10.1074/jbc.M116.758300

**Published:** 2017-02-06

**Authors:** Kaveesha J. Wijesinghe, Sarah Urata, Nisha Bhattarai, Edgar E. Kooijman, Bernard S. Gerstman, Prem P. Chapagain, Sheng Li, Robert V. Stahelin

**Affiliations:** From the ‡ Department of Chemistry and Biochemistry, The Eck Institute for Global Health and The Boler-Parseghian Center for Rare and Neglected Diseases, University of Notre Dame, Notre Dame, Indiana 46556,; the § Department of Medicine, University of California, San Diego, La Jolla, California 92093-0652,; the Departments of ¶ Physics and; ** Biomolecular Sciences Institute, Florida International University, Miami, Florida 33199,; the ‖ Department of Biological Sciences, Kent State University, Kent, Ohio 44242, and; the ‡‡ Department of Biochemistry and Molecular Biology, Indiana University School of Medicine-South Bend, South Bend, Indiana 46617

**Keywords:** Ebola virus, hydrogen-deuterium exchange, mass spectrometry (MS), oligomerization, phosphatidylserine, plasma membrane, Filovirus, Marburg Virus, VP40, viral budding

## Abstract

Marburg virus (MARV) is a lipid-enveloped virus from the *Filoviridae* family containing a negative sense RNA genome. One of the seven MARV genes encodes the matrix protein VP40, which forms a matrix layer beneath the plasma membrane inner leaflet to facilitate budding from the host cell. MARV VP40 (mVP40) has been shown to be a dimeric peripheral protein with a broad and flat basic surface that can associate with anionic phospholipids such as phosphatidylserine. Although a number of mVP40 cationic residues have been shown to facilitate binding to membranes containing anionic lipids, much less is known on how mVP40 assembles to form the matrix layer following membrane binding. Here we have used hydrogen/deuterium exchange (HDX) mass spectrometry to determine the solvent accessibility of mVP40 residues in the absence and presence of phosphatidylserine and phosphatidylinositol 4,5-bisphosphate. HDX analysis demonstrates that two basic loops in the mVP40 C-terminal domain make important contributions to anionic membrane binding and also reveals a potential oligomerization interface in the C-terminal domain as well as a conserved oligomerization interface in the mVP40 N-terminal domain. Lipid binding assays confirm the role of the two basic patches elucidated with HD/X measurements, whereas molecular dynamics simulations and membrane insertion measurements complement these studies to demonstrate that mVP40 does not appreciably insert into the hydrocarbon region of anionic membranes in contrast to the matrix protein from Ebola virus. Taken together, we propose a model by which association of the mVP40 dimer with the anionic plasma membrane facilitates assembly of mVP40 oligomers.

## Introduction

Marburg virus (MARV)^3^ belongs to the Filoviridae family like its cousin the Ebola virus (EBOV) (1). These viruses cause hemorrhagic fever in human and non-human primates causing high fatality rates, which have reached as high as 90% in some outbreaks (2, 3). To date, there are no FDA approved drugs to treat EBOV or MARV infection, despite several therapeutic approaches employed during the 2013–2016 EBOV outbreak in West Africa (4).

EBOV and MARV are lipid-enveloped viruses and their lipid envelope is extracted from the host cell plasma membrane when the nascent viral particles bud out from the host cell (5). The matrix protein VP40 is a peripheral protein located beneath the lipid envelope providing structural stability to the viral particle and a protein matrix layer to facilitate budding of virions from the plasma membrane. Expression of VP40 as the only filovirus protein in mammalian cells is sufficient to form filamentous structures referred to as viral like particles (VLPs) that closely resemble the authentic viruses (6, 7). MARV VP40 (mVP40) is able to recruit MARV glycoprotein (GP), which is a transmembrane protein found on the viral envelope, to sites of budding (8). mVP40 is also able to interact with the nucleocapsid of the virus, which encapsulates the negative sense RNA genome of the virus (9). Phosphorylation of mVP40 at specific tyrosine residues also facilitates recruitment of the nucleocapsid into filopodia or mVP40-enriched sites of budding (10).

Recent crystal structure determination of mVP40 found that like Ebola VP40 (eVP40), mVP40 exists as a dimer and contains a N-terminal (NTD) with an α-helical dimer interface and a C-terminal domain (CTD) that mediates membrane binding (11). In addition to the eVP40 dimer, eVP40 forms other oligomeric states, which have been crystalized (12). Through hydrophobic CTD-CTD interactions eVP40 dimers can further assemble into hexamers that have been shown to be crucial for viral matrix assembly and budding (12). eVP40 dimers were shown to assemble into hexamers via the CTD-CTD interface as well as a NTD interface that is somewhat buried in the dimer. A conformational change is hypothesized to occur in eVP40 monomers or dimers, which would exclude the monomers at the ends of the hexamer, displacing the CTD from the NTD revealing the oligomerization site.

This conformational displacement exposes a residue, which was much more buried in the dimer, Trp^95^. Trp^95^ forms the integral part of the NTD oligomerization interface along with Glu^160^. These residues allow the NTDs to assemble the central core of the hexamer. These rearranged hexamers further assemble into long filamentous structures that form the matrix layer and facilitate the budding process (12). Significant oligomerization of eVP40 was hypothesized to occur at the plasma membrane as an anionic membrane mimetic, dextran sulfate, induced this VP40 structural rearrangement (12). In agreement with this hypothesis, VP40 hexamers and larger oligomers have been observed at the plasma membrane inner leaflet where the matrix assembly and budding take place (13–15). Apart from the hexamer, eVP40 also forms an octameric ring structure that binds RNA and this RNA interaction with the eVP40 octamer was shown to be important for the viral life cycle (12, 16). To date, only the mVP40 dimer structure has been solved and thus much less information is available on how mVP40 may oligomerize in the presence of membranes.

The NTD of mVP40 is similar to eVP40 sharing 42% sequence identities and similar folds. The NTD-NTD dimer interface interactions are shown to be important for viral assembly and budding as mutation of threonine (Thr^105^) at the dimer interface prevented mVP40 assembly, budding of VLPs, and greatly reduced anionic membrane binding (11). The key residues involved in NTD oligomerization in eVP40 hexamers were also found to align in sequence and structure with mVP40, Trp^83^ and Asn^148^, respectively. Mutation of this Trp and Asn in mVP40 greatly reduced VLP budding (11). The CTD of mV40 is significantly different from eVP40. The basic patch of mVP40 features a flatter and more extended surface than that of eVP40, and consists of basic amino acids Lys^210^, Lys^211^, Arg^215^, Lys^218^, Lys^259^, Lys^264^, Lys^265^, and Arg^266^. Mutations of basic patch residues abrogated mVP40 assembly and greatly reduced VLP budding (11). The significant reduction in assembly is most likely due to the significant reduction in anionic lipid binding by these mutations (11). We have previously investigated membrane binding properties of mVP40 and found that consistent with the structural features of the mVP40 basic patch, mVP40 associates with the plasma membrane via nonspecific electrostatics highly dependent on the anionic charge density of the membrane (17). However, the consequences of lipid binding on mVP40 assembly and structural transitions is still unknown.

To investigate the origins of phosphatidylserine (PS) or phosphatidylinositol 4,5-bisphosphate (PI(4,5)P_2_) binding and structural consequences of PS or PI(4,5)P_2_ binding by mVP40, we have used hydrogen/deuterium exchange mass spectroscopy (HDX-MS) to measure the deuteration level of the mVP40 amino acid sequence with and without lipids. HDX-MS has been previously used to investigate protein structure, conformational changes, and dynamics of ligand binding (18–20). HDX-MS has also been successfully used to investigate structural changes occurring on peripheral membrane proteins that interact with cell membranes (21, 22). Results reported herein demonstrate robust coverage of the mVP40 sequence with respect to deuterium exchange and reveals the primary sites of PS and PI(4,5)P_2_ binding and structural transitions necessary to expose the conserved NTD interface involved in mVP40 oligomerization. Lipid binding assays, molecular dynamics (MD) simulations, and membrane insertion measurements were used to complement the HDX-MS work and elucidate the mechanism by which mVP40 associates with the plasma membrane.

## Results

### mVP40 coverage map of pepsin fragmentation

**Figure 1.**
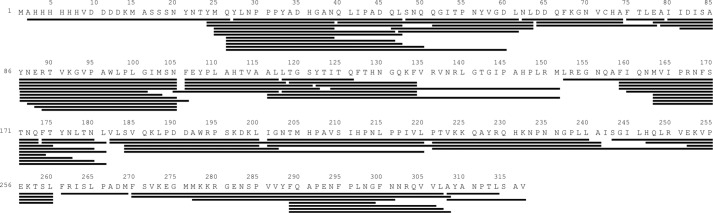
**Peptide coverage map of mVP40.** Pepsin digestion produced 77 unique overlapping peptides that resulted in 99% coverage of the mVP40 sequence.

To determine the optimum digestion conditions that yielded the best peptide fragmentation pattern for mVP40, changes in the denaturant (GdnHCl) concentration (0, 0.08, 0.8, 1.6, 3.2, and 6.4 m) were employed. The most significant coverage of unique peptides was found at 0.8 m GdnHCl (final concentration in HD exchange sample = 0.5 m), where 77 unique peptides were selected and analyzed for the data generation. (The coverage map for these 77 peptides is shown in Fig. 1.)

### Deuterium on-exchange of mVP40 in solution

**Figure 2.**
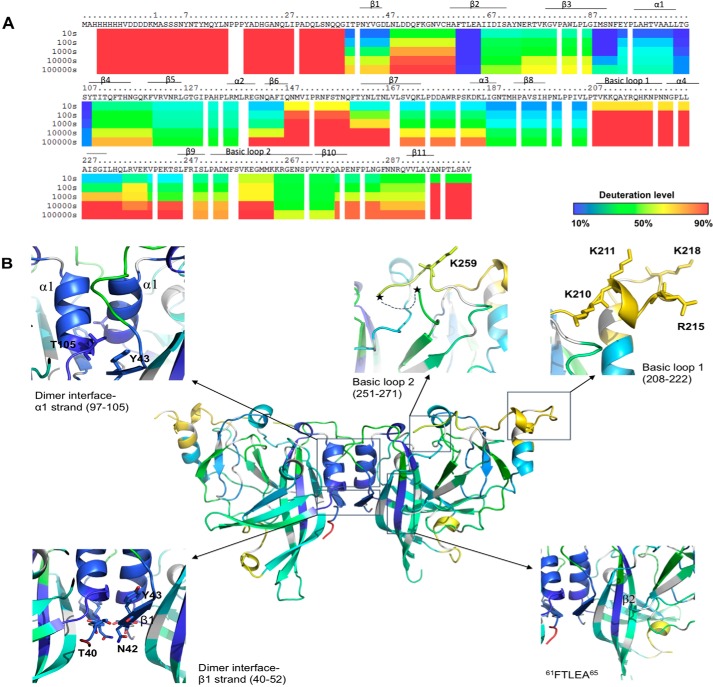
**Hydrogen deuterium exchange of mVP40 in the absence of lipid vesicles.** *A*, ribbon map indicating the percentage of hydrogen/deuterium exchange that occurred in peptide fragments over the entire exchange period. Each *row* corresponds to each time point from 10 to 100,000 s. Color coding indicates the percentage of deuterium incorporation at the given time point. *B*, percent labeling of the 10-s time point was mapped onto the crystal structure of mVP40 (PDB code 5B0V).

The percentage of hydrogen-deuterium exchange for each unique peptide used in the analysis was determined at five different time points (10, 100, 1000, 10,000, and 100,000 s) and is depicted in the ribbon map of mVP40 (Fig. 2, *A* and *B*). The first 14 residues at the N terminus are part of the His tag sequence used in mVP40 purification and exhibited significant deuteration likely due to their accessibility to the solvent. Note that these residues are not shown in the structural figures as they were not resolved in the X-ray crystal structure. The first 38 residues of the NTD of mVP40 are also highly flexible and did not appear in the crystal structure of the protein. The deuterium incorporation to these residues (as well as the aforementioned His tag) was more than 90% even at the 10-s exchange period (Fig. 2*A*). Residues from 40 to 52 of the β1 strand of mVP40 are part of the NTD-NTD dimer interface (see Fig. 2*B*) (11) and at the 10-s exchange time period deuterium incorporation was very low (∼10% deuteration level). This is consistent with the crystal structure of mVP40, which elucidated three direct H-bonds that occur across the dimeric interface between Thr^40^ and Asn^42^, Thr^40^ and Tyr^43^, and between Asn^42^ and Asn^42^ of the opposing monomers (11). These close contacts and H-bonds may limit solvent accessibility to this region lowering the deuterium accumulation as shown in Fig. 2. As the HD exchange time increases, the amount of deuterium incorporated also increases. This suggests the region of the β1 strand is dynamic and may undergo structural fluctuations over prolonged time periods, which facilitate exposure of amide hydrogens of that region.

The α1 helix, mVP40 residues Ala^97^-Thr^105^ (see Fig. 2, *A* and *B*) are also a part of the dimer interface (11) and demonstrated slow HD exchange levels throughout the exchange time periods analyzed. The residues of α1 helical dimer interface exchanged much more slowly even at prolonged exchange time periods when compared with residues from the β1 strand (residues 40–52) (Fig. 2, *A* and *B*). The α1 helices form multiple hydrophobic contact points and therefore largely exclude water. Also, the α helical structures have most of their amide hydrogens involved in internal hydrogen bonding interactions, which can slow HD exchange rates. As a result of impaired solvent accessibility to this region and slow HD exchange rates, deuterium accumulation in this region is very low.

The CTD of mVP40 contains a broad basic patch, which has been shown to mediate membrane binding to anionic lipids such as phosphatidylserine (17). The basic patch primarily consists of two basic loops where the first basic loop (Thr^208^-Asn^222^, Fig. 2, *A* and *B*) harbored about 70–80% deuterium incorporation level at the 10-s exchange period and >90% deuterium incorporation at the 100-s time point and beyond. These residues are solvent exposed in the crystal structure and their amide hydrogens are not part of secondary structures and not extensively H-bonded. Therefore, residues of this basic loop, in the absence of membranes, exhibit >90% deuterium incorporation.

The second basic loop of the CTD spans from Leu^251^ to Pro^271^ (Fig. 2, *A* and *B*) (11). However, at the 10-s exchange period this region exhibited 50% deuterium incorporation and took ∼10,000 s to reach considerable deuteration levels unlike the first basic loop (Fig. 2, *A* and *B*). In the crystal structure of mVP40, the short disordered loop that contains residues Lys^264^, Lys^265^, and Lys^266^ was not resolved (delineated by * in Fig. 2*B*). It was surprising to observe that this disordered loop, which lacks secondary structure and is likely to be solvent exposed exhibited slower deuterium incorporation levels. However, it is possible that although disordered, the amide hydrogens of this region participate in internal H-bonding producing slower exchange rates. Indeed, MD simulations of the mVP40 protein demonstrated that basic residues in this loop are involved in intermolecular interactions, which may limit exchange with deuterium (see more discussion below).

An interesting observation was made regarding the ^61^FTLEA^65^ sequence (Fig. 2, *A* and *B*) of the β2 strand of mVP40. This region showed very low levels of deuterium incorporation (∼10%) even at the longest time point (100,000 s) of HD exchange (Fig. 2, *A* and *B*). The β2 strand forms an antiparallel β sheet with the β3 and β7 strands and due to strong H-bond networks in antiparallel β sheets, we can expect the HD exchange rates to be low. Also, its location between β strands causes this region to be buried inside the mVP40 NTD, minimizing its solvent accessibility. As a result, even after a prolonged period of incubation with D_2_O, deuterium incorporation did not significantly occur in this region. Thus, we hypothesize if mutations were to occur in the β2 strand that increased the flexibility or hydrophilicity of this region, it would most likely destabilize the NTD.

### Effects of phospholipid membrane interaction on mVP40 basic loops

To resolve the effects of phosphatidylserine binding on mVP40 structural dynamics, HD exchange experiments were done on mVP40 following preincubation with phospholipid vesicles. We hypothesized this approach could confirm the mVP40 membrane binding surface that has been previously established (11, 17) and elucidate structural transitions, revealing sites of mVP40 oligomerization that occur during viral matrix assembly. We employed lipid vesicles that were composed of POPC:POPS (55:45) as a model phospholipid membrane as previously employed in mVP40 lipid binding assays (17). mVP40 exhibits saturation levels of membrane binding in the presence of ∼40 mol % POPS (17). By providing an excess of anionic phospholipids at 45 mol % POPS, we are ensuring mVP40 is achieving its saturation level of binding. Exchange experiments were carried out at five different time points from 10 to 100,000 s to directly compare deuterium incorporation of mVP40 incubated in solutions without lipids.

**Figure 3.**
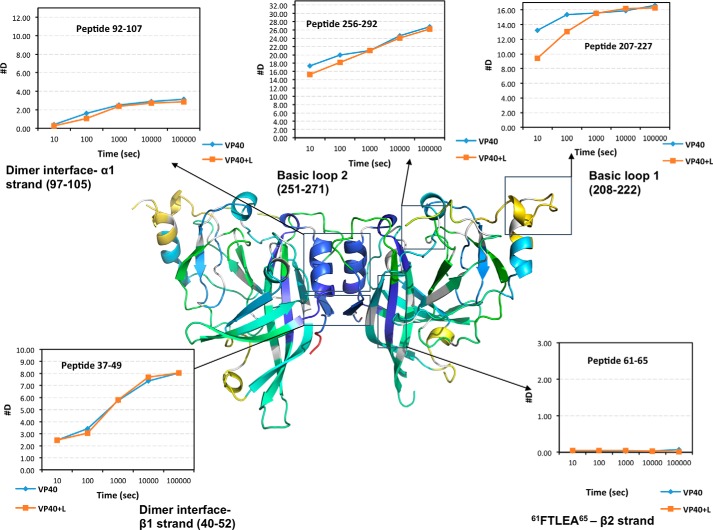
**Deuterium accumulation plots generated for mVP40 alone and mVP40 in the presence of lipid vesicles.** Deuterium level at each time point is plotted for mVP40 alone (*dark blue line*), mVP40 + lipid vesicles (*orange line*) for each unique peptide identified using DXMS explorer software.

To obtain peptide specific HD exchange information, deuterium accumulation plots for both mVP40 alone and mVP40 in the presence of POPC/POPS vesicles were generated. Examples of such deuterium accumulation plots are shown in Fig. 3. We first looked at the differences in deuterium incorporation in the basic patch region of mVP40 in the presence of liposomes compared with the protein alone sample.

In the presence of PS containing liposomes, basic loop 1 (208–222) exhibited a reduction in deuterium incorporation into the peptide backbone compared with protein alone at 10- and 100-s time points. However, as longer incubation times were analyzed a similar level of deuterium incorporation was observed for mVP40 with and without PS. Because basic loop 1 of mVP40 associates with the PS containing membranes (11), the basic patch is likely shielded from the bulk solvent by membranes, causing reduced deuterium incorporation. However, the interaction of mVP40 with PS containing membranes is likely to be very dynamic with continuous association and dissociation events occurring facilitating deuterium exchange to occur with the bulk solution when mVP40 transiently dissociates from the membrane. As a result, at longer periods of D_2_O incubation, more deuterium may get incorporated to the basic patch 1 region. Basic patch 2 (251–271) exhibited a similar trend as basic patch 1 at short incubation times with PS containing vesicles (10 s and 100 s), although to begin with the level of deuterium incorporation in the absence of liposomes was moderate (∼50%).

**Figure 4.**
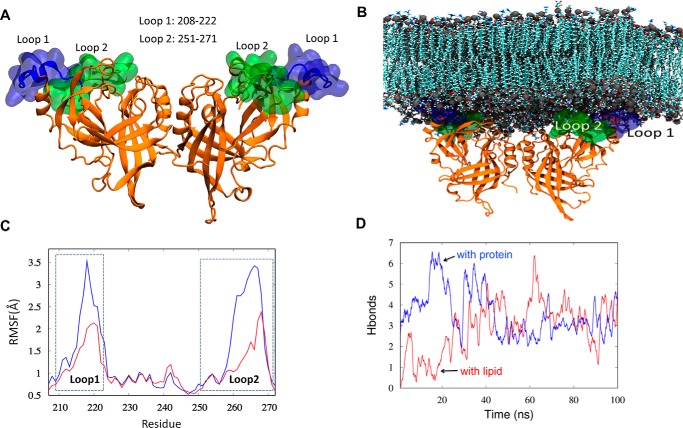
**Modeling mVP40 interaction with the plasma membrane.** *A*, structure of the mVP40 dimer following modeling of missing residues from the crystal structure into the *in silico* system. The basic loop 1 is highlighted in *blue* and basic loop 2 is in *green. B*, mVP40 dimer associated at the lower leaflet of the plasma membrane (snapshot after 100 ns of MD simulation). *C*, root mean square fluctuations (*RMSF*) for the CTD segment containing residues in basic loop 1 and basic loop 2. *Blue* is mVP40 without lipid and *red* is mVP40 in the presence of the membrane bilayer. *D*, the number of hydrogen bonds (h-bonds) for loop 2 residues (Lys^264^, Lys^265^, and Arg^266^) (*blue*, mVP40 protein-protein h-bonds; *red*, mVP40 h-bonds with lipid) as a function of time (moving average, averaged every 100 ps). Initially, these residues make more h-bonds with protein but later make about the same with protein and lipids. On average, ∼6 h-bonds are made at all times (3 with other amino acids, 3 with lipids).

In an attempt to further understand the observed differences in deuteration pattern between basic loop 1 and basic loop 2, which can equally cause strong disruptions in proteins ability to bind to membranes as well as form VLPs in cells (11) we used MD simulations to model interactions of the mVP40 basic patch with a phospholipid membrane. Residues that were absent on the crystal structure of protein, including the missing basic loop residues can be modeled into the *in silico* system allowing us to extract useful information on its location on the surface of the protein as well as its membrane interactions (Fig. 4).

**Figure 5.**
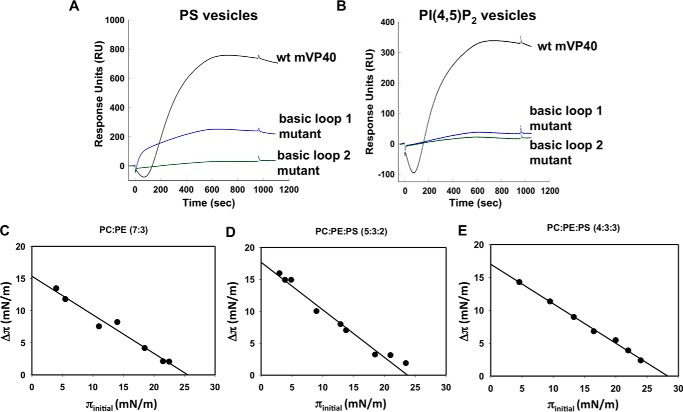
**mVP40 SPR lipid binding and monolayer penetration assays.** *A* and *B*, comparison of phospholipid binding ability of two basic patch mutants to wild type mVP40. R215A/K218A is a basic loop 1 mutant and K264A/K265A/R266A is a basic loop 2 mutant. SPR sensograms are shown for 500 nm wild type mVP40 (*black*), R215A/K218A (*blue*), K264A/K265A/R266A (*green*) for: *A,* POPC:POPS (60:40); *B*, PC:PI(4,5)P_2_ (92.5:7.5) liposomes. Both mutants exhibit significant reduction in vesicle interaction for both lipid compositions in comparison to wild type mVP40. *C–E*, change in surface pressure *versus* initial lipid monolayer surface pressure is plotted following interaction with mVP40. Phospholipid compositions tested for mVP40 insertion are shown *above* each graph. MIP values are obtained by extrapolating to the *x* axis (x intercept). MIP values for PS containing monolayers were below 30 mN/m.

Fig. 4*A* shows the initial structure of the mVP40 dimer, with basic loop 1 and basic loop 2 highlighted in surface representations. During the MD simulation of the mVP40-membrane system we were able to observe the mVP40 basic patch interacting with the membrane. Fig. 4*B* shows a snapshot of the protein-membrane system at 100 ns of the MD trajectory. We evaluated the flexibility of the residues composing the basic patch in the presence and absence of the membrane system by calculating the root mean square fluctuations, as shown in Fig. 4*C*. The CTD segments that included basic loop 1 and basic loop 2 exhibited significantly reduced fluctuations when they interact with the membrane (Fig. 4*C*). Also the degree of reduction we observe for both basic loop 1 residues and basic loop 2 residues are similar in magnitude suggesting both of these loops are engaging with the membrane in similar intensities. Indeed, quantitative assessment of binding to lipid vesicles using surface plasmon resonance (SPR) demonstrated mutation of basic loop 1 or basic loop 2 greatly reduced the ability of mVP40 to associate with liposomes containing 40 mol % PS or 7.5 mol % PI(4,5)P_2_ (Fig. 5, *A* and *B*).

Interestingly, basic loop 2 residues displayed a slower exchange rate with deuterium than basic loop 1 residues (Fig. 2*A*) in the absence of lipids. This suggests basic loop 2 may undergo more significant intramolecular interactions prior to engaging the lipid membrane, which would shield amide hydrogens and slow the exchange. MD simulations revealed that several residues in basic patch 2 engaged in intramolecular h-bonds. This included the Lys^264^ side chain with the Gly^261^ backbone, the Lys^264^ side chain with the Lys^259^ backbone, the Lys^265^ side chain with the side chain of Asp^177^, the Lys^265^ backbone with the backbone of Val^258^, and the Arg^266^ side chain interacting with the Asp^177^ side chain and backbone of Met^263^. Throughout the MD simulations, basic loop 2 residues increased h-bonds with lipids while also maintaining intramolecular h-bonds for an average of ∼6 h-bonds for basic loop 2 residues throughout the trajectory (Fig. 4*D*). The major interactions of basic loop 2 with the membrane included the side chain of Lys^264^ and PS, the side chain of Lys^265^ and PS, and the side chain of Arg^266^ and PI.

### Effects of phospholipid membrane interaction on mVP40 NTD dimer interface

Deuterium accumulation levels in the α1 helix (97–105) and the β1 strand that composed the dimer interface of the mVP40 dimer showed no significant differences in the presence and absence liposomes (Fig. 3). Under both conditions HD exchange was low, resulting in low deuterium incorporation suggesting this region has been largely inaccessible to bulk solvent. Because the deuterium accumulation levels were unchanged for mVP40 in the presence of liposomes, strongly suggesting that the dimer does not dissociate into monomers upon membrane association and/or dissociation and the dimer is likely to be the building block of other oligomeric forms of mVP40.

The ^61^FTLEA^65^ sequence of the β2 strand of mVP40 that was solvent inaccessible in the absence of liposomes also showed no change in deuteration levels in the presence of liposomes. Therefore, it is unlikely that the anti-parallel β sheet it forms with β3 and β7 strands does become more solvent exposed during mVP40 membrane interactions or oligomerization triggered during matrix assembly.

### HDX-MS of mVP40 reveals potential oligomerization interfaces

**Figure 6.**
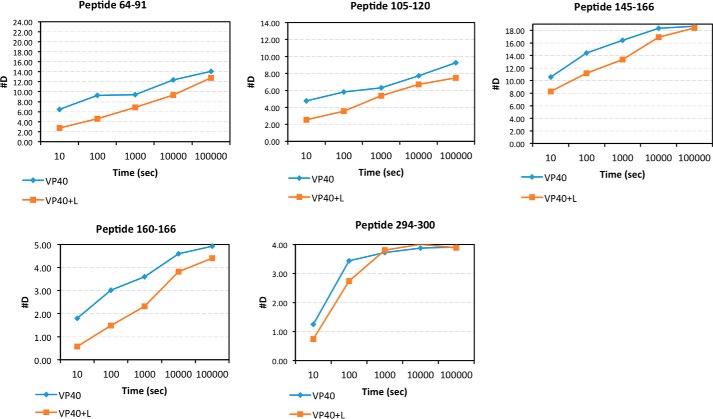
**Effects of phospholipid membranes on the HD exchange pattern of mVP40.** Deuterium accumulation plots of peptide fragments of mVP40 that showed significant differences in the deuterium levels in the presence and absence of liposomes. Deuterium levels of mVP40 alone in the absence of lipid vesicles is shown in *light blue line*; VP40+L denotes mVP40 in the presence of PS containing lipid vesicles (*orange line*).

**Figure 7.**
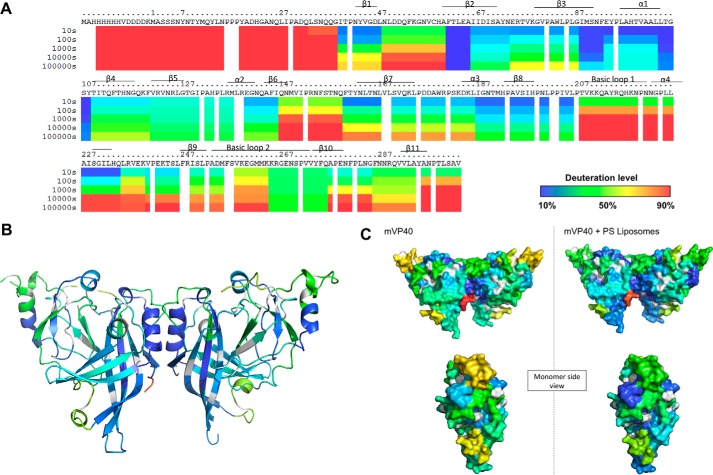
**Hydrogen/deuterium exchange of mVP40 in the presence of PS containing vesicles.** *A*, ribbon map indicating the percentage of hydrogen/deuterium exchange that occurred in peptide fragments over the entire exchange period. Each *row* corresponds to each time point from 10 to 100,000 s. Color coding indicates the percentage of deuterium incorporation in the given time point. *B*, percent labeling of the 10-s time point mapped onto the crystal structure of mVP40 (PDB code 5B0V). Color coding is as indicated in *A. C*, space filling models of mVP40 protein (*right*) and mVP40 after incubation with lipids. Side view of a mVP40 monomer is also shown for both conditions.

Some of the deuterium accumulation plots of peptide fragments showed significant changes in the deuteration levels in the presence of phospholipid membranes compared with the protein alone sample (Fig. 6). These peptide fragments do not originate from the dimer interface or basic patch of mVP40 and hence we hypothesize that these peptides arise from regions of mVP40 that undergo structural changes triggered by mVP40 interaction with phospholipid membranes. For further analysis of these regions we produced a rainbow map for mVP40 in the presence of membranes representing the percentage of hydrogen/deuterium exchange for each overlapping peptide for the entire length of reaction (Fig. 7*A*).

Compared with the mVP40 protein sample without lipids, the HD exchange pattern of mVP40 in the presence of PS differ considerably. Overall, in the presence of PS, mVP40 exhibited slower HD exchange rates than that of protein alone sample indicating significant regions of less solvent accessibility (Fig. 7, *A–C*). The reduction of HD exchange levels in the basic patch region of mVP40 was anticipated because membrane binding would shield the previously solvent-exposed surface. However, the reduction of HD exchange levels were not limited to the basic patch regions, signaling that association of phospholipid membrane at the C-terminal basic patch triggers other structural changes on mVP40. If the protein undergoes oligomerization and forms filamentous structures, the oligomerization sites would become solvent inaccessible. Also, if the filamentous structures further assemble to form the viral matrix it will cause greater shielding of individual mVP40 dimers lowering HD exchange levels.

Alternatively, low HD exchange levels in the presence of liposomes may also result if the protein can penetrate the membrane forming hydrophobic interactions with the lipid acyl chains of the membrane. This can result in reduced solvent accessibility of the protein domain that penetrates into the membrane. To ensure the observed reduction in HD exchange levels of mVP40 in the presence of negatively charged liposomes is not due to protein insertion into the membrane we did monolayer insertion experiments (Fig. 5, *C* and *D*). Maximum insertion pressure (MIP) of the protein that can be derived from these experiment is useful to determine whether the protein can insert into the membrane at a given lipid composition. MIP values below 30 mN/m suggests the protein does not penetrate into the hydrocarbon region of the phospholipid membrane (23). We measured the change in surface pressure induced by mVP40 on lipid monolayers composed of 0, 20, or 30 mol % PS, however, the MIP values derived from these experiments did not exceed 30 mN/m. This confirmed that mVP40 does not significantly penetrate into PS containing membranes. Hence, this further supports the hypothesis that regions of mVP40 exhibiting significant reductions in deuteration levels (excluding the basic patches and dimer interface) are most likely due to membrane triggered structural changes including oligomerization in mVP40.

**Figure 8.**
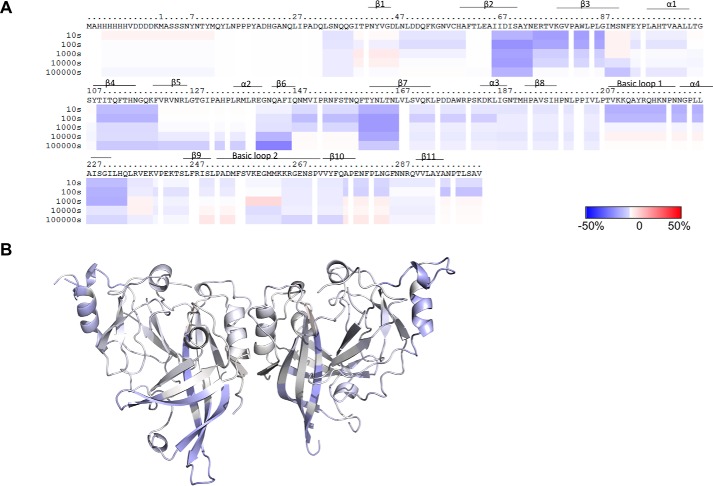
**Influence of membrane association on mVP40 as a measure of the change in deuteration level (D%) in comparison to mVP40 in the absence of liposomes.** *A*, difference in deuteration percentage of mVP40 in the presence of liposomes was mapped to the sequence over the entire exchange period. Each *row* corresponds to each time point from 10 to 100,000 s. Color coding: *blue* indicates the regions that exchange slower in the presence of lipid and *red* indicates the regions that exchange faster in the presence of lipid. *B*, differences in deuteration level (D%) in the presence of liposomes of the 10-s time point mapped on to the crystal structure of the mVP40 (PDB code 5B0V).

**Figure 9.**
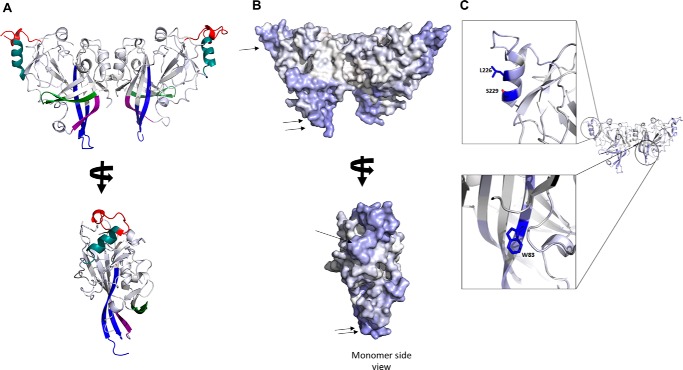
**Regions of mVP40 that have undergone significant changes in deuteration percentage (D%) triggered by PS-dependent membrane association.** *A*, major D% changes mapped to the mVP40 crystal structure. In *blue,* Ile^66^-Gly^87^; *purple,* Phe^160^-Leu^167^; *green*, Thr^109^-Phe^120^; *red*, Thr^208^-Asn^222^; *teal*, Gly^223^-Leu^235^. *B*, space filling model of mVP40 at the 10-s time point showing regions on the mVP40 structure that has shown significant changes in the D%. *Single arrows* indicate the α4 helix, which may provide CTD-CTD oligomerization surface. *Double arrows* indicate a potential oligomerization interface at the NTD that gets exposed following structural rearrangement of mVP40 following membrane association. *C*, residues Leu^226^, Ser^229^ of the α4 helix in the CTD and W83 in the NTD may mediate key interactions during oligomerization.

By comparing ribbon maps generated for mVP40 protein alone sample and mVP40 preincubated with PS containing membranes, the change in the deuteration percentage (%D) of mVP40 for the entire length of the experiment was mapped (Fig. 8). This data provides a clear overview of the regions of the mVP40 protein that have shown significant differences in the deuteration level in response to the presence of PS. We identified locations of the peptide sequences that showed significant changes in deuteration by mapping them to the crystal structure of mVP40 (Fig. 9). Among the peptide fragments that showed significant changes in deuteration were Ile^66^ to Gly^87^, Thr^109^ to Phe^120^, Phe^160^ to Leu^167^, the basic loop 1 region (Thr^208^-Asn^222^), and Gly^223^ to Leu^235^ (the α4 helix) (Fig. 9*A*).

Basic loop 1 (Thr^208^-Asn^222^) undergoes significant reduction in deuterium levels as it interacts with anionic membranes as a result of reduced solvent exposure of this region. Interestingly, we also find that the α4 helix located beneath the basic loop 1 also harbors significant reduction in deuteration levels. The sequence of α4 helix shows hydrophobic character and we hypothesize the loss of accessibility of the solvent may be due to assembly of mVP40 dimers in an end to end manner to form the hexamer, which further assembles into filamentous structures. Notably, a similar mechanism of filament assembly was recently proposed for eVP40 (12).

**Figure 10.**
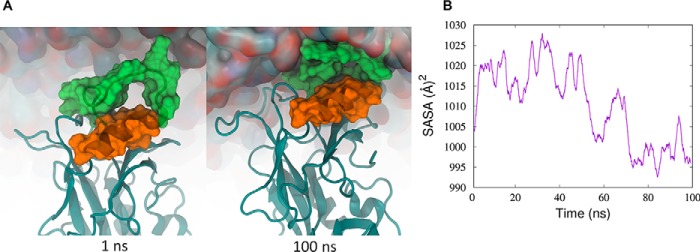
**Conformational rearrangement of basic loop 1 (residues 208–221) due to membrane interactions.** *A*, as basic loop 1 interacts with the membrane, the gap between the α-helix below loop 1 is slightly reduced. *B*, change in the solvent accessible surface area of α4 helix residues (223 to 231) along the MD trajectory. At the end of 100 ns of MD simulation, the solvent accessible surface area of the α4 helix was reduced.

An alternative explanation to the observed changes in deuteration levels in the α4 helix is that during interaction of mVP40 with the membrane, the α4 helix gets shielded by the basic loop 1 as the loop adjust its position to form electrostatic interactions with the membrane. MD simulations of mVP40 in the presence of membrane show that as the basic loop 1 interacts with the anionic membrane surface, it slightly envelops part of the α4 helix below loop 1 (Fig. 10*A*). The solvent accessible surface area of the α4 helix shows a slight decrease after about 50 ns of MD trajectory (Fig. 10*B*). This may also explain why lower deuteration levels were observed for the α4 helix apart from an oligomerization event.

### HDX-MS of mVP40 in the presence of zwitterionic membranes and PI(4,5)P_2_ containing membranes

We broadened our investigation of the engagement of basic patch residues of mV40 with phospholipid membranes by experimenting on how mVP40 interacted with membranes devoid of anionic lipid (POPC zwitterionic lipid) as well as membranes containing another important anionic plasma membrane lipid, PI(4,5)P_2_.

**Figure 11.**
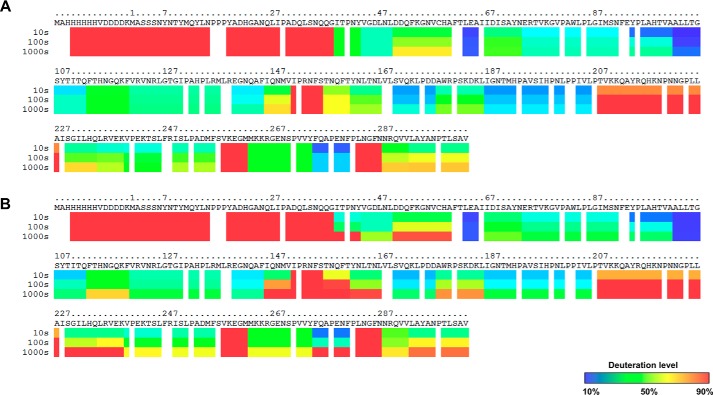
**Hydrogen/deuterium exchange of mVP40 in the presence and absence of zwitterionic lipid vesicles.** *A*, ribbon map indicating the percentage of hydrogen/deuterium exchange that occurred in peptide fragments over the entire exchange period for the protein alone sample. *B*, ribbon map indicating the percentage of hydrogen/deuterium exchange that occurred in peptide fragments over the entire exchange period for the protein preincubated with PC vesicles. Each *row* corresponds to each time point from 10 to 1000 s. Color coding indicates the percentage of deuterium incorporation in the given time point. Residue numbering begins after the first 14 amino acids that compromise the His tag. *B*, percent labeling of the 10-s time point mapped on to the crystal structure of the mVP40 (PDB code 5B0V).

**Figure 12.**
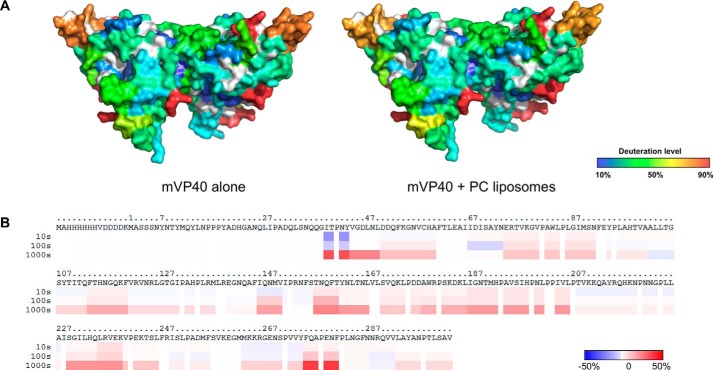
**Influence of presence of zwitterionic phospholipid membranes on mVP40 as a measure of the change in deuteration level (D%) in comparison to mVP40 in the absence of zwitterionic membranes.** *A*, percent deuterium labeling of mVP40 in the presence and absence of PC vesicles for the 10-s time point mapped on to the crystal structure of the mVP40 (PDB code 5B0V). *B*, difference in deuteration percentage of mVP40 in the presence of PC liposomes is mapped to the sequence over the entire exchange period. Each *row* corresponds to each time point from 10 to 1000 s. Color coding: *blue* suggests the regions that exchange slower in the presence of PC vesicles; and *red* suggests the regions that exchange faster in the presence of PC vesicles.

**Figure 13.**
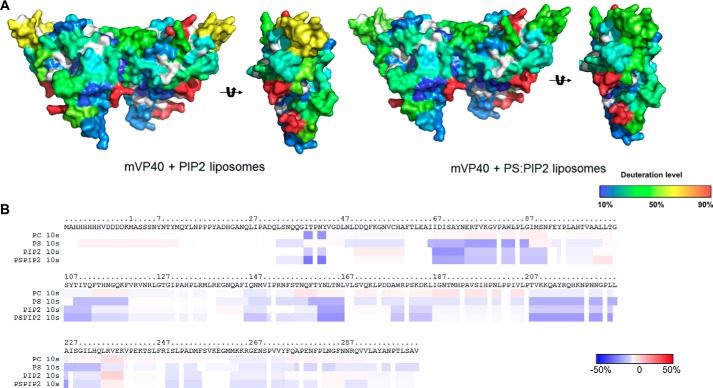
**mVP40 interaction with PI(4,5)P_2_ containing vesicles results in a similar hydrogen/deuterium exchange pattern compared with PS containing vesicles.** *A*, space filling models of mVP40 protein after preincubation with PI(4,5)P_2_ vesicles (*right*) and PS + PI(4,5)P_2_ combined vesicles (*left*). Side view of a mVP40 monomer is also shown for both conditions. *B*, difference in deuteration percentage of mVP40 mapped to the sequence over the 10-s exchange period for all lipid compositions tested. Color coding: *blue* suggests the regions that exchange slower in the presence of liposomes; and *red* suggests the regions that exchange faster in the presence of liposomes.

We have previously shown mVP40 to be an anionic charge sensor that exclusively interacts with negatively charged lipids (17). We have hypothesized that for the structural changes in mVP40 necessary for oligomerization to occur, the mVP40 dimer must first form interactions with the phospholipid membrane. Therefore, we performed several other HDX-MS experiments where we preincubated mVP40 protein with lipid vesicles that were composed entirely of zwitterionic phospholipid/phosphatidylcholine. In the absence of an anionic membrane surface, mVP40 cannot form significant electrostatic interactions with membranes and hence does not bind zwitterionic membranes at nanomolar or micromolar concentrations (17). In support of the central hypothesis, the HDX pattern of mVP40 protein alone and mVP40 in the presence of PC vesicles looked very similar at 10- and 100-s time points, indicating the protein exhibited no significant changes in the solvent accessibility in the presence of PC liposomes (Figs. 11 and 12*A*). This is in stark contrast to the HDX-MS pattern observed when mVP40 was preincubated with PI(4,5)P_2_ or PS vesicles, which caused significant reduction in solvent accessibility (Fig. 13*B*). We also observed that at the 1000-s time point, mVP40 exhibited higher deuterium incorporation compared with the protein alone sample suggesting an increase in the solvent accessibility of the protein in the presence of neutral phospholipid membrane (Figs. 11*B* and 12*B*). This can be a result of nonspecific zwitterionic interactions causing the mVP40 protein to slightly deform its structure making some regions more accessible to solvent than in the absence of PC. However, further studies are necessary to understand this occurrence and its significance including more HDX-MS data at longer time points (10,000 s and 100,000 s) especially to see if the trend continues.

Next, we investigated the effect of having another anionic phospholipid in the membranes on mVP40 solvent accessibility. PI(4,5)P_2_ is another anionic phospholipid that is found at the inner leaflet of the plasma membrane and plays a significant role in recruitment of peripheral proteins to the plasma membrane surface (24). PI(4,5)P_2_ harbors a net negative charge of −4 at pH 7.4 (25). From our previous findings, we know that mVP40 exhibited saturation binding when the membranes contained 7.5% (mol %) of PI(4,5)P_2_ (17). We also tested a lipid composition that contained 20% (mol %) of PS and 5% (mol %) PI(4,5)P_2_ to observe the combined effects on mVP40 of two different types of anionic phospholipids in the membrane. This composition also previously facilitated saturation binding of mVP40 to phospholipid membranes (17). For both membrane compositions, the changes in the deuteration labeling of the protein looked very similar to each other and to the labeling pattern of mVP40 in the presence of 45% (mol %) PS (Fig. 13, *A* and *B*). The changes in the deuteration percentage for the basic patch residues looked very similar under all conditions with the exception of more deuterium incorporation in basic loop 1 for PI(4,5)P_2_ compared with PS or PS + PI(4,5)P_2_ (Fig. 13). This confirmed the lack of specific binding pockets on the mVP40 surface that promote stereospecific interaction among specific anionic phospholipid head groups further confirming mVP40 exhibits nonspecific interactions with anionic phospholipids as an electrostatic charge sensor.

## Discussion

The majority of deuteration level changes triggered by membrane association were detected in the NTD of mVP40 (Fig. 8, *A* and *B*). This included regions spanning from the end of the β2 strand to the end of β3 strand (Ile^66^-Gly^87^), the entire β4 strand, and the beginning of β5 strand (Thr^109^ to Phe^120^ includes), as well as the β7 strand (Phe^160^-Leu^167^). The NTD of eVP40 was particularly important in oligomerization of rearranged hexamers following eVP40 conformational changes induced by a membrane mimetic as described previously (12). β2, β3, and β7 strands together form an antiparallel β sheet structure and we found the concentration of two major deuteration level changes in this region is intriguing. We hypothesize that this β sheet structure provides an oligomerization interface for the mVP40 NTD following association with PS containing membranes. This is based upon the aforementioned HD exchange data but also the homology of this region in mVP40 to that of eVP40.

The structural transition of the eVP40 NTD would make the previously buried Trp^95^ residue, which forms the core of the oligomerization surface of eVP40, more accessible to protein-protein interactions. Sequence alignment between eVP40 and mVP40 reveals that Trp^83^ of mVP40 aligns with the Trp^95^ of eVP40 (12). Trp^83^ residue is present in the β3 strand that undergoes significant reduction in solvent accessibility. Therefore, it is possible to suspect this β2, β3, and β7 antiparallel β sheet structure provides an oligomerization surface for matrix assembly. The β5 strand is also likely to be part of the same oligomerization surface as it also extends in the same direction as the β2, β3, and β7 antiparallel β sheet structure. Moreover, three other mVP40 residues have been shown to be conserved with the eVP40 NTD oligomerization interface including Arg^136^, Arg^139^, and Asn^148^. In the case of Asn^148^, a significant reduction in deuteration level following incubation with PS containing membranes was detected (Figs. 7–9).

**Figure 14.**
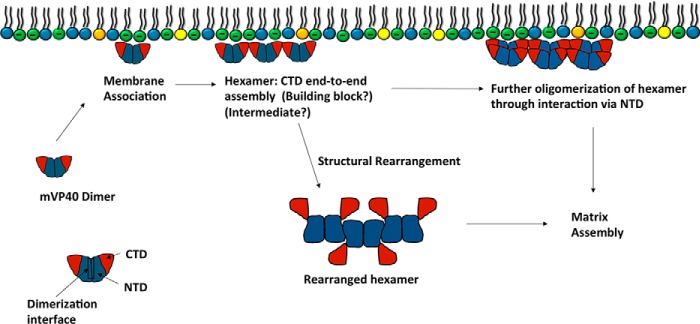
**Hypothesized oligomerization pathways of mVP40 that leads to viral matrix assembly inside an infected host cell.** Cytosolic mVP40 dimers associate with the inner leaflet of the plasma membrane by interacting with anionic phospholipids such as phosphatidylserine. mVP40 dimers further oligomerize on the membrane surface by interacting via the CTD in an end-to-end manner to form a linear hexamer. Matrix assembly may occur through further oligomerization of linear hexamers with another hexamer unit by interacting via the NTD of composing dimers that facilitate the formation of linear filaments. Alternatively, the linear hexamer may be an intermediate that undergoes structural rearrangement similar to the eVP40 protein. The rearranged hexamer may then assemble into the matrix layer via NTD interactions.

To the best of our knowledge, this is the first structural study done on mVP40 using HDX-MS. Here we have optimized conditions to get robust coverage of deuterium exchange on the mVP40 amino acid sequence, which has identified structural changes triggered by mVP40 interaction with anionic membranes. In Fig. 14 we propose a model of mVP40 assembly in the presence of PS containing membranes. Here, the mVP40 dimer associates with PS containing membranes with basic patches enriched with several cationic residues (Lys^210^, Lys^211^, Arg^215^, and Lys^218^ in basic loop 1; Lys^264^, Lys^265^, and Arg^266^ in basic loop 2), mutation of which previously abrogated anionic lipid binding, plasma membrane localization, and virus-like particle formation (11). Quantitative binding measurements performed in this study confirmed the important roles of basic patch 1 and 2 where mutations of these patches associated weakly at nanomolar concentrations compared with wt mVP40. MD simulations further provided molecular insight demonstrating both basic loop 1 and 2 engage the anionic membrane surface with electrostatic and h-bonds throughout the trajectory time.

Following association with the anionic membrane interface, mVP40 oligomerizes through a NTD interface that was partially buried and solvent inaccessible in the absence of PS. VP40 oligomers are also likely to oligomerize through the α4 helix in the CTD in a similar manner to CTD end-to-end contacts previously shown for eVP40 (12). Although this helix does tilt toward the membrane interface, reducing its solvent accessibility, the modest decrease in solvent accessibility uncovered by MD simulations was not as significant as the drastic changes to this helix in deuterium incorporation in the presence of anionic membranes.

Strikingly, mVP40 membrane penetration in the presence of PS containing membranes was significantly less than that previously found for eVP40 (14, 26). Hydrophobic interactions were found to be a critical component of eVP40 assembly where PS-dependent membrane penetration of eVP40 was essential to eVP40 oligomerization and formation of VLPs (14, 26). mVP40 instead seems to rely on a dense cationic CTD surface and multiple interactions with the anionic membrane surface (17) to effectively assemble and oligomerize. This further underscores the differential lipid binding mechanisms that have been proposed for mVP40 and eVP40 (11, 12, 15, 17, 27, 28), which harbor membrane binding CTDs of modest sequence identity (16% identical). In closing, HDX-MS combined with *in vitro* biochemistry and cellular studies should be an excellent tool to further reveal assembly properties of filovirus matrix proteins.

## Experimental procedures

### Materials

1-Palmitoyl-2-oleoyl-*sn*-glycero-3-phosphocholine (POPC), 1-palmitoyl-2-oleoyl-*sn*-glycero-3-phospho-l-serine (POPS), 1,2-dioleoyl-sn-glycero-3-phospho-, and 1′-myo-inositol 4,5-bisphosphate (PI(4,5)P_2_) were purchased from Avanti Polar Lipids (Alabaster, AL) and used without further purification. D_2_O was obtained from (Cambridge Isotope Laboratories Inc., Tewksbury, MA). Formic acid and GdnHCl were purchased from (Fisher Scientific).

### Protein expression and purification

mVP40 protein harboring a His_6_ tag was expressed and purified as described by Wijesinghe *et al.* (17).

### Optimization of peptide digestion

Before deuterium-labeling experiments were performed, optimum digestion conditions that provide a optimum peptide fragmentation pattern with overlapping peptides that produce the maximum coverage the entire length of protein had to be selected (24). Various digestion conditions were tested by varying the concentration of GdnHCl (0, 0.08, 0.8, 1.6, 3.2, and 6.4 m) in quenching buffer that also contained 0.8% (v/v) formic acid at pH 2.3 and 16.6% (v/v) glycerol. 3 μg of protein was used in each experiment. Quenching experiments were carried out as described under “Deuterium Exchange Experiments.”

### Preparation of multilamellar vesicles (MLVs)

For the preparation of PS containing liposomes, POPS and POPC were mixed in the ratio of 45:55, respectively, to form the MLVs. PI(4,5)P_2_ containing liposomes were prepared by mixing POPC and PI(4,5)P_2_ in a 92.5:7.5 molar ratio, whereas combined PS:PI(4,5)P_2_ MLVs were prepared by mixing POPC, POPS, and PI(4,5)P_2_ at 75:20:5 molar ratio. Briefly, lipids were mixed in the indicated molar ratio and dried under N_2_ gas for 10 min. The dried lipid film was hydrated using dH_2_O at 65 °C for 15 min. Following hydration, the lipid film was vortexed vigorously to form MLVs (17, 29). For on exchange experiments, 3 μg of protein was incubated with excess MLVs for 20 min at room temperature. Final lipid concentration was 2.8 mm and final volume was 6 μl. For protein alone samples, 3 μg of protein was diluted in dH_2_O.

### Deuterium exchange experiments

HDX experiments were initiated by mixing 18 μl of D_2_O buffer (8.3 mm Tris, pH 7.2, containing 150 mm NaCl) into 6-μl samples at pH 7.2. Following addition of D_2_O buffer, samples were incubated 10, 100, 1000, 10,000, and 100,000 s at 4 °C. The deuterium exchange was quenched at 0 °C for 1 min by the addition of 6 μl of optimized quenching buffer (0.8% formic acid, 2.5 m GdnHCl, 0.6 m NaH_2_PO_4_, and 50% glycerol, pH 2.4). The control samples for correcting back exchange were also prepared as previously described (25). The samples were then immediately frozen on dry ice and stored at −80 °C until used in mass spectrometry analysis.

### Pepsin digestion and LC-MS analysis of samples

Pepsin digestion and LC-MS analysis was done as described previously (30, 31). Briefly, frozen samples were loaded onto a cryogenic autosampler and then were thawed for 60 s at 5 °C before being loaded onto an immobilized pepsin column (16-μl bed volume) at a flow rate of 20 μl/min to allow digestion of proteins. Pepsin-generated peptide fragments were collected on a C18 trap column (0.2 × 1 mm, Optimize Tech Inc., Oregon City, OR) for desalting, and flowed through a reverse phase C_18_ column (0.2 × 50 mm, MAC-MOD Analytical Inc., Chadds Ford, PA) for fragment separation using a linear acetonitrile gradient (6.4–38.4%) over 30 min. The eluent was directed into the OrbiTrap Elite mass spectrometer (Thermo Fisher Scientific) for MS analysis. Peptide identification was done by using Proteome Discoverer software (Thermo Fisher Scientific) (30, 31).

### Deuterium quantification and data analysis

The centroid values of all the deuterated peptides were calculated with DXMS Explorer (Sierra Analytics Inc., Modesto, CA), and then converted to the amount of deuterium incorporation with corrections for back-exchange (26). All HDX data were normalized to 100% D_2_O content with an estimated average deuterium recovery of 75%. The deuterium incorporation within the sequence of VP40 was further sublocalized with an in-house MATLAB program using the difference between overlapping peptides as previously described (27). The standard deviation of HDX measurements performed on our system is normally less than 2% of the mean of triplicate experiments (28).

### Molecular dynamics simulations on mVP40 membrane interactions

The mVP40 structure was obtained from the Protein Data Bank (PDB code 5B0V) and the missing residues were added with Modeler (32). The protein and plasma membrane system was built using Charmm-GUI membrane builder web interface (33). The asymmetric lipid bilayer was generated with the lipid distributions of POPC, POPE, POPS, POPI, sphingomyelin (PSM), and cholesterol comparable with the known plasma membrane composition (34, 35). The composition of different lipid molecules in the lower leaflet of the plasma membrane were in the ratio of 11:33:18:9:7:20 (POPC:POPE:POPS:POPI:PSM:cholesterol) and the resulting membrane contained 574 lipids. The membrane-protein system was solvated with TIP3 water in cubic boxes and neutralized with 0.15 m KCl. The final solvated system contained more than 200,000 atoms. A separate system for the mVP40 dimer was set up without the membrane that also contained more than 200,000 atoms.

All-atom MD simulations were performed with CHARMM36 force field (36) using NAMD 2.11 (37). The particle mesh Ewald method (38) was used to calculate the long range ionic interactions. For each system, a 10,000-step minimization was performed followed by equilibration runs. For the membrane system, a six-step equilibration (as given by Charmm-GUI) was performed using 1- or 2-fs time steps. This was followed by 100-ns NPT production run (constant pressure/temperature) at 300 K using 2-fs time step. The pressure was controlled using the Nose-Hoover Langevin-piston method. Similarly, the temperature was controlled using the Langevin temperature coupling with a friction coefficient of 1 ps^−1^. The VMD software (39) was used to visualize and analyze the simulations.

### Lipid monolayer insertion experiment

Monolayer insertion experiments were performed as described in Ref. 40. Briefly, a circular Teflon trough was filled with Tris buffer (10 mm Tris, 150 mm KCl, 0.2 mm EDTA at pH 7.2). Total volume was 6.5 ml. The phospholipid monolayer was formed on the air/water interface by adding phospholipids of the mentioned compositions dissolved in chloroform:methanol (2:1) solvent in a dropwise manner using a 10-μl Hamilton syringe. Following establishment of a desired initial surface pressure, chloroform was allowed to evaporate before introducing mVP40 protein into the subphase by injecting through a port on the trough. Concentration of protein introduced to the subphase was 0.12 μm. The change in the surface pressure with time was measured using a Platinum Wilhelmy plate and a PS4 Nima surface pressure sensor. The increase of the surface pressure following addition of the protein was plotted as a function of initial surface pressure of the monolayer. MIP value was obtained by extrapolating to the *x* axis (23, 40).

### Surface plasmon resonance binding assays

All SPR experiments were performed as previously described in Refs. 41 and 42. Briefly, a POPC:POPS (60:40) active surface and a POPC control surface or a POPC:PI(4,5)P_2_ (92.5:7.5) active surface and a POPC control surface was used to determine the saturation response for the wild type and two mutant mVP40 proteins at 500 nm concentrations.

## Author contributions

K. J. W., S. L., and R. V. S. conceived of and designed the studies. K. J. W., S. U., N. B., P. P. C., and S. L. performed the experiments and analyzed the data. E. E. K. oversaw and directed monolayer penetration data collection and analysis, whereas B. S. G. and P. P. C. oversaw and directed molecular dynamics data collection and analysis. K. J. W. and R. V. S. wrote the paper with input from all authors.

## Footnotes

3 The abbreviations used are:

MARV: Marburg virus
EBOV: Ebola virus
HD: hydrogen/deuterium
HDX: hydrogen/deuterium exchange
VLP: viral-like particle
NTD: N-terminal domain
CTD: C-terminal domain
PS: phosphatidylserine
eVP40: Ebola VP40
mVP40: Marburg VP40
PI(4,5)P_2_: phosphatidylinositol 4,5-bisphosphate
GdnHCl: guanidine hydrochloride
SPR: surface plasmon resonance
MIP: maximum insertion pressure
mN: milliNewton
POPC: 1-palmitoyl-2-oleoyl-*sn*-glycero-3-phosphocholine
POPS: 1-palmitoyl-2-oleoyl-*sn*-glycero-3-phospho-l-serine
MLV: multilamellar vesicle
PDB: Protein Data Bank
POPI: 1-palmitoyl-2-oleoyl-*sn*-glycero-3-phosphoinositol
POPE: 1-palmitoyl-2-oleoyl-*sn*-glycero-3-phosphoethanolamine.
